# Out-of-Pocket Costs and Perceived Financial Burden Associated with Prostate Cancer Treatment in a Quebec Remote Area: A Cross-Sectional Study

**DOI:** 10.3390/curroncol28010005

**Published:** 2020-11-30

**Authors:** Abir El-Haouly, Anais Lacasse, Hares El-Rami, Frederic Liandier, Alice Dragomir

**Affiliations:** 1 Département des Sciences de la Santé, Université du Québec en Abitibi-Témiscamingue (UQAT), Rouyn-Noranda, QC J9X 5E4, Canada; abir.el-haouly@uqat.ca (A.E.-H.); anais.lacasse@uqat.ca (A.L.); 2 Centre Hospitalier de Rouyn-Noranda, Centre Intégré de Santé et de Services Sociaux de l’Abitibi-Témiscamingue (CISSS-AT), Rouyn-Noranda, QC J9X 2A9, Canada; hares_rami@hotmail.com (H.E.-R.); fliandier@hotmail.com (F.L.); 3 Department of Surgery, Division of Urology, Faculty of Medicine, McGill University, Montreal, QC H3G 1A4, Canada; 4 Research Institute, McGill University Health Centre, Montreal, QC H3G 1A4, Canada

**Keywords:** prostate cancer, costs and cost analysis, cost-of-illness, out-of-pocket costs, health expenditures, perceived financial burden, remote area

## Abstract

*Background*: In publicly funded healthcare systems, patients do not pay for medical visits but can experience costs stemming from travel or over-the-counter drugs. We lack information about the extent of this burden in Canadian remote regions. This study aimed to: (1) describe prostate cancer-related out-of-pocket costs and perceived financial burden, and (2) identify factors associated with such a perceived burden among prostate cancer patients living in a remote region of the province of Quebec (Canada). *Methods:* A cross-sectional study was conducted among 171 prostate cancer patients who consulted at the outpatient clinic of the Centre Hospitalier de Rouyn-Noranda. *Results:* The majority of patients (83%) had incurred out-of-pocket costs for their cancer care. The mean total cost incurred in the last three months was $517 and 22.3% reported a moderate, considerable or unsustainable burden. Multivariable analysis revealed that having incurred higher cancer-related out-of-pocket costs (OR: 1.001; 95%CI: 1.001–1.002) private drug insurance (vs. public, OR: 5.23; 95%CI: 1.13–24.17) was associated with a greater perceived financial burden. Having better physical health-related quality of life (OR: 0.95; 95%CI: 0.913–0.997), a university education (vs. elementary/high school level, OR: 0.03; 95%CI: 0.00–0.79), and an income between $40,000 and $79,999 (vs. ≤ $39,999, OR: 0.15; 95%CI: 0.03–0.69) were associated with a lower perceived burden. *Conclusion*: Prostate cancer patients incur out-of-pocket costs even if they were diagnosed many years ago and the perceived burden is significant. Greater attention should be paid to the development of services to help patients manage this burden.

## 1. Introduction

Prostate cancer is the second most common cancer diagnosis among Canadian men [1,2]. According to the International Agency for Research on Cancer (IARC), prostate cancer incidence in Canada was equal to 128.8 per 100,000 persons in 2018, twice that of lung and colorectal cancer [1]. The widespread uptake of prostate-specific antigen screening in 1993 has led to increased detection rates in younger men of early-stage localized prostate cancer [3]. As shown by the 95% five-year survival rate, most men with prostate cancer survive for many years [2]; the prevalence of this disease is therefore high. In fact, in 2018, the IARC reported a five-year prevalence of 414.9 per 100,000 in Canada [1]. Although men with prostate cancer are living longer, they live with cancer treatment side effects, such as sexual, urinary and bowel problems [4]. Thus, prostate cancer leads to a significant burden for patients [5], suggesting that this pathology represents an economic burden for those affected. These costs are expected to rise for several reasons such as an increased incidence, early detection, treatment of low-risk cases, use of expensive new therapies, and an aging population [5]. The economic burden of prostate cancer may therefore become greater, especially for older men, frequently affected by prostate cancer [6] and already facing human and economic frailty [7].

The economic burden of illness is defined in terms of direct medical costs (e.g., hospitalization, physician fees, etc.), direct non-medical costs (e.g., traveling), and loss of productivity [8]. In Canada, direct medical costs are generally covered by public healthcare insurance [9,10]. However, services provided to patients outside hospitals and clinics (e.g., psychotherapy, physiotherapy, etc.) are often not covered [9,10]. Such costs may be reimbursed by private health insurance plans if patients have this type of insurance, either paid by the employer or by themselves. Otherwise, they must be paid out-of-pocket [9,10]. In addition, patients are usually responsible for direct non-medical costs since they are not covered by insurance [11]. Depending on the magnitude and duration of the out-of-pocket costs, these can be perceived by patients and their caregivers as a significant financial burden [12].

The out-of-pocket costs and perceived financial burden of prostate cancer have been reported in various studies across the world [13,14,15,16,17,18,19,20,21,22,23,24]. Gordon et al. (2015) showed that, in Australia, among incidents and prevalent cases of prostate cancer, out-of-pocket costs in the three-month period before the study averaged AUD 9205; these costs caused a “great deal” of distress for 20% of patients [13]. In Canada, few studies have focused on this specific topic [17,18,19,20,21]. Longo et al. (2006) showed that in the one-month period leading up to the study enrolment, out-of-pocket costs paid by Ontario cancer patients, including prostate cancer patients, equaled $585; 20% of these patients perceived this financial burden as significant or unmanageable [19]. To our knowledge, no study has been conducted on this subject in a remote area in Canada. In such areas, access to certain treatments, offered only in large centers, is challenging since it is conditional on travelling over several hundred kilometers, and certain costs may not be covered by the healthcare system. It should also be noted that long-term out-of-pocket expenses for prostate cancer patients are rarely studied in North America (most studies focus on patients who have not yet received treatment or who are undergoing treatment) [14,17,18,19,25]. Estimating prostate cancer out-of-pocket costs and assessing the perceived financial burden in the specific context of remote areas is thus important, especially to provide healthcare professionals with evidence allowing them to inform patients of these costs when discussing treatment.

Our study aimed to: (1) describe prostate cancer-related out-of-pocket direct medical and non-medical costs, (2) describe patients’ perceived financial burden; and (3) identify clinical and sociodemographic factors associated with such a burden among prostate cancer patients living in a remote region of the province of Quebec (Canada).

## 2. Methods

### 2.1. Study Setting and Sample

The remote regions of Quebec (*n* = 6) are those removed from major urban centers, on the eastern, northern and western borders of Quebec [26]. Abitibi-Témiscamingue is one such region [26], where 58% of the population lives in urban poles and 42% in rural areas [27]. To receive treatments not offered in the region, such as external or internal radiotherapy, patients must travel approximately 417 to 867 km. Between November 2017 and February 2019, a cross-sectional cost-of-illness study was conducted among adults with prostate cancer from the Outpatient Clinic of the Hôpital de Rouyn-Noranda of the Centre intégré de santé et de services sociaux (CISSS) de l’Abitibi-Témiscamingue, which covers the entire regional population of 147,508 individuals [28]. At the end of their medical visit, patients were informed of the study by urologists (*n* = 2) in the order of their arrival. To be eligible, patients had to be able to answer the questionnaire in French. They were also to be free of cognitive or physical problems preventing them from responding to the study questionnaire. Those who were interested in receiving more information about the study provided contact information and left the clinic with a package containing an information letter, the questionnaire, and a consent form to share information contained in their medical records. Patients who decided to participate in the study completed the questionnaire at home and returned it in a prepaid envelope included in the package. If we did not receive the envelope one week after the medical visit, we called patients to enquire about their willingness to participate. There were no financial incentives. All patients with prostate cancer were included in the sample, regardless of the time elapsed since diagnosis and treatment. Therefore, we captured a broad cross-section of cancer survivors. This study was approved by the Ethics Committees of the Université du Québec en Abitibi-Témiscamingue (2015-05-El-Haouly, A.) and of the CISSS des Laurentides (which acts as a research ethics committee on behalf of the CISSS de l’Abitibi-Témiscamingue) (2016-349-É).

### 2.2. Questionnaire and Variables

Study variables were collected using a standardized, self-administered questionnaire. Before the study, this questionnaire was piloted among 10 prostate cancer patients to verify its clarity. No changes were required; the respondents understood and completed it easily in under 40 min.

To estimate the financial burden of prostate cancer, we considered the direct medical and non-medical costs associated with managing this disease from a patient perspective (out-of-pocket costs). These costs were assessed for the three months preceding the survey, using questions based on the Longo et al. study [19]. Patients were asked to report costs related to prostate cancer and out-of-pocket expenses: (1) travel expenses for hospitalizations and medical visits, parking fees, accommodation expenses not covered by the public healthcare insurance; (2) travel expenses for visits to other healthcare professionals (e.g., psychologist), parking fees, accommodation expenses and consultation fees; (3) purchase of over-the-counter and prescription drugs; (4) purchase of vitamins, supplements, devices and equipment; (5) complementary and alternative medicine (e.g., homeopathy); (6) homemaking services (e.g., cleaning); (7) family care (elder or child); (8) healthcare services (nursing, physiotherapy, psychotherapy); (9) in-home healthcare services (e.g., nursing); (10) accommodations/meals; and (11) other costs. Personal vehicle transportation costs over the last three months were calculated based on the travel distance to the hospital/clinic (kilometers), multiplied by the number of trips made during this period, and multiplied by provincial rates allowed by the Canada Revenue Agency to calculate travel expenses for 2018 (i.e., $0.55 per kilometer) [29]. For all other travelling, the transportation costs were equal to the ticket price paid for transportation by bus, taxi, or plane. We also evaluated costs according to time since the first treatment. Costs were calculated in Canadian dollars. Furthermore, participants were asked to report the number of days of absence from work for themselves as well as their friends and family during the last three months.

The perceived burden associated with out-of-pocket costs was assessed using a six-point Likert scale inspired by the Longo et al. study [19] (“not a burden,” “light burden,” “moderate burden,” “considerable burden, but sustainable,” “considerable burden that is difficult to manage and stressful,” “unsustainable burden”). Based on the distribution of our results, a categorization was achieved according to the reporting of a moderate/considerable/unsustainable burden (yes/no). 

Generic health-related quality of life was measured using the French-Canadian version of the 12-Item Short Form Health Survey-version 2.0 (SF-12v2) [30]. This shorter version of SF-36 includes 12 questions and provides two summary measures representing the physical and mental components of health-related quality of life. Scores range from 0 to 100, where a higher score corresponds to a better quality of life. Scores on each summary scale were calculated with standard scoring algorithms and normalized using general population values (mean = 50; SD = 10). This scale has good psychometric properties (test-retest reliability of the physical summary component (0.86 to 0.89); test-retest reliability for the mental component (0.76 to 0.77)) [30].

Finally, the questionnaire included treatments received (i.e., active surveillance, radical prostatectomy, radiotherapy (internal and external), chemotherapy and hormonotherapy). The calendar dates of treatments received were also requested, allowing the calculation of time elapsed since the first treatment using the calendar date of the questionnaire completion. Sociodemographic information, including age, race, education, relationship status, annual family income, professional status, region of residence, and level of insurance coverage, was collected. Comorbidities were also evaluated using the Self-Administered Comorbidity Questionnaire [31]. Clinical characteristics at diagnosis, including the Gleason score, prostate-specific antigen (PSA) level and clinical cancer stage, were abstracted from medical records.

### 2.3. Statistical Analysis

Data analyses were conducted using IBM SPSS Statistics, version 22.0 (Armonk, NY, USA: IBM Corp.). Descriptive analyses were used to assess patient characteristics, out-of-pocket costs, and the perceived financial burden. To identify factors associated with perception of a moderate/considerable/unsustainable financial burden (yes/no), a multivariate logistic regression model was built. Time elapsed since first treatment and all other variables that had a *p* < 0.15 in univariate analyses were included in the final multivariate model. A total of 10 variables were entered into the model. “Cost*income” and “cost*drug insurance” interactions were tested and were found to be non-significant and were not included in the final multivariate model. Variance inflation factors were used to rule out any multicollinearity problems. In all our analyses, *p* < 0.05 was considered statistically significant.

## 3. Results

**Figure 1:**
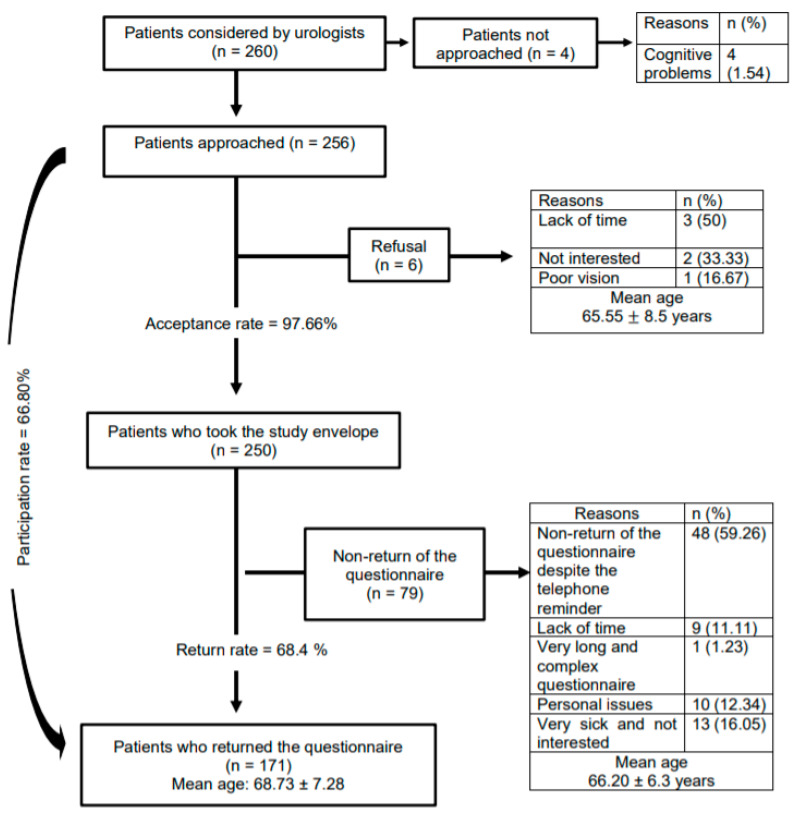
Study flow chart.

During the recruitment period, 256 patients were consecutively approached to participate in this study; 171 of them returned the self-administered questionnaire (68.40%). The recruitment flow diagram can be found in Figure 1. Table 1 summarizes the sociodemographic and clinical characteristics of the population. Overall, patients were predominantly living with a partner (83.6%), had a public drug insurance plan (71.9%), were retired or not working (74.3%), had not completed post-secondary education (57.3%), and had an annual family income between $20,000 and $39,999 (51.5%). Their mean age was 68.73 ± 7.28 years. At diagnosis, most patients had a clinical tumor stage ≤ T2a (76.5%) and a Gleason score of 7 (52.6%).

On average, patients received their first treatment 4.11 ± 3.73 years (min: 0, max: 20, median: 3) before questionnaire completion. The most frequently received first treatment after diagnosis was radiotherapy (a total of 57.14% participants). As this treatment was not available in the participants’ region of residence, this was received outside their region. About one quarter of patients had received as a first treatment radical prostatectomy (26.2%), 13.7% were subjected to active surveillance, 2.36% had undergone hormonotherapy and 0.6% had received another treatment. 

### 3.1. Out-of-Pocket Costs

**Figure 2:**
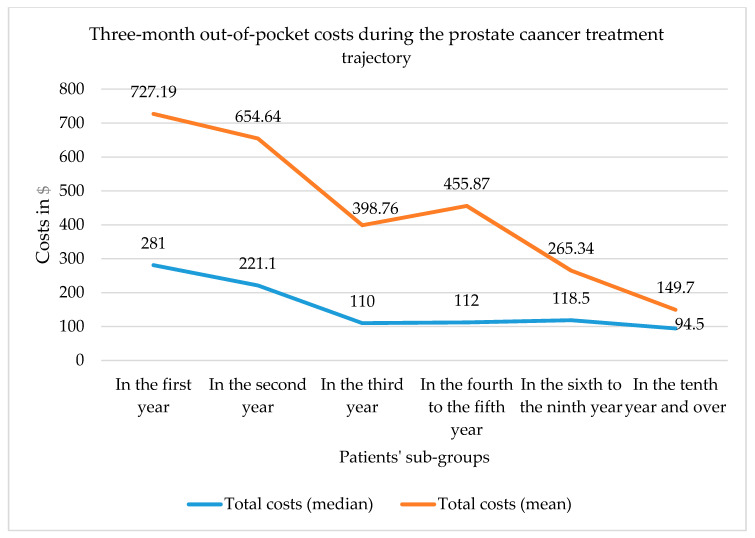
Out-of-pocket costs according to time elapsed since first treatment.

Most patients (*n* = 142; 83%) reported out-of-pocket costs in the last three months. Such costs are displayed in Table 2 for the whole study sample, the subgroup of patients with non-null costs and for three subgroups according to the time elapsed since the first treatment (one of patients in their first year, one of patients in their second year, and one of patients in their third year or more). Among the whole study sample, the mean total cost was $516.19 (SD: 1029.71; median: 139). Travel costs were the highest type of out-of-pocket cost (mean: $344.19; SD: 808.53; median: 88). Out-of-pocket costs varied between $727.19 (one year or less since first treatment) and $149.7 (10 years or more since first treatment) (Figure 2). Figure 2 also shows that costs declined rapidly from the second year onward, that the average cost continued to decline each year, and that the median cost showed stability from the third year onward.

### 3.2. Absences from Work

Some of the patients in our study were not working (*n* = 129; 75.4%). Among the 42 working men, 21 (50%) had experienced cancer or treatment-related days of absence from work during the last three months. The mean for absenteeism was 30 days ± 37.06. Most patients (38.01%) took their days of absence as paid workdays, 33.33% took time off without pay and 28.56% used vacation or leave days. 

### 3.3. Perceived Financial Burden and Associated Factors

Participants reported their perception of the financial burden resulting from their cancer-related out-of-pocket expenses: 22.3% reported a moderate/considerable/unsustainable burden (moderate: 12.3%; considerable, but sustainable: 7%; considerable, difficult to manage and stressful: 1.8%; unsustainable: 1.2%). Table 3 presents the results of univariate and multivariate models conducted to identify factors associated with the perceived financial burden. Controlling for time elapsed since treatment, the multivariate analysis revealed that higher out-of-pocket costs (OR: 1.001; 95%CI: 1.001–1.002) or having a private drug insurance (vs. public, OR: 5.23; 95%CI: 1.13–24.17) was associated with a greater likelihood of reporting a moderate, considerable or unsustainable financial burden. Patients with better physical health-related quality of life (OR: 0.954; 95%CI: 0.913–0.997), a university education (vs. elementary and high school level, OR: 0.03; 95%CI: 0.00–0.79), or an income between $40,000 and $79,999 (vs. ≤ $39, 999, OR: 0.15; 95%CI: 0.03–0.69) were less likely to report a moderate, considerable or unsustainable financial burden.

## 4. Discussion

To our knowledge, this is the first Canadian study to provide an estimate of prostate cancer costs from the perspective of patients living in remote areas, and an assessment of the perceived economic burden. We found that most participants had incurred out-of-pocket costs in the three months leading up to the study. These costs primarily involved travel expenses and could be incurred throughout treatment and even 10 years later. This study also shows that half of working patients had experienced an average of 30 days of absence from work in the last 90 days, one third of which as unpaid leave. In addition, our results suggest that almost one quarter of patients perceived out-of-pocket costs as burdensome and that independent factors associated with this burden include higher out-of-pocket costs, private drug insurance, lower annual family income, lower education, and poorer physical health-related quality of life.

### 4.1. Out-of-Pocket Costs

In this study, the average out-of-pocket costs related to prostate cancer amounted to $516.19 per patient with high variability (SD: $1029.71; range: $0–$6148). These costs are lower than those estimated by Longo et al. [19] (one-month costs of $585, or $1755 per three months, assuming that costs were similar across all three months). The explanation for this is threefold. First, the study investigated costs among rural and urban patients whose use of healthcare services (complementary and alternative medicine) was high, probably because of their availability. Second, Longo’s sample included women with breast cancer whose expenditure was found to be higher than those of other cancer patients, likely to be due to their age and propensity to use more healthcare services. Third, Longo’s study investigated costs among patients with recently diagnosed cancers and it is known that costs are higher in the first year of diagnosis and treatment [33]. The prostate cancer patients in our study lived in remote locations, made almost no use of complementary and alternative medicine, and, on average, had received their first treatment four years prior. Although costs assessed in our study are lower, they do, however, remain substantial.

We calculated the proportion of income consumed by out-of-pocket costs for prostate cancer care. For households earning under $40,000 per year (51.5% of our sample), these costs represent 5.2% of their income. This situation is problematic as the Canadian healthcare system claims to provide universal healthcare for medically necessary healthcare services provided on the basis of need, rather than the ability to pay [34]. This situation would justify a change in the allocation of health resources that could optimize healthcare coverage.

Of the total costs, the highest out-of-pocket cost was related to travel ($344.19 ± $808.53). Consistent with this finding, Longo et al. [18] showed that cancer patients undergoing treatment paid $372 for travel in addition to other costs ($213). Radiotherapy was the treatment most frequently received by the participants in our sample. Receiving radiotherapy entails travelling at least 417 km, which may explain why travel costs were the highest in our study. Despite their public insurance program, the financial aid offered by the Ministry of Health and Social Services of Quebec to patients who must travel outside the region to receive treatment, and the free shuttle bus offered by the CISSS de l’Abitibi-Témiscamingue to users who travel to Gatineau to receive radiotherapy treatment, Canadian patients living in the remote area of Abitibi-Témiscamingue still report out-of-pocket costs. It is therefore relevant to develop additional services and policies to help these patients manage the economic burden of prostate cancer.

One of the unexpected results of our study is that patients incurred cancer-related out-of-pocket costs up to 10 years following their first treatment. In their Ontario-based study, Oliveira et al. argued that long-term prostate cancer survivors have incurred costs for their cancer care (the sample included patients 2 to 13 years following diagnosis) [12]. Prostate cancer costs are therefore long-term expenses which may be related to the management of long-term adverse effects caused by treatment, including impotence and impaired urinary and/or bowel function [35]. Accordingly, prostate cancer patients pay out-of-pocket expenses both at the time of treatment and while dealing with the sequelae of treatment. However, as demonstrated by Jayadevappa et al. [14], costs decrease in the second year, likely to be due to the higher costs of the initial treatment phase compared to the follow-up phase [36]. Despite this downward trend, these costs are not inconsequential for already frail elderly people [7]. It is therefore important to inform newly diagnosed patients of this ensuing economic burden (e.g., leaflets, video clips, smartphone apps or through the nurse case manager) to help prepare them for its management through budget planning.

### 4.2. Absence from Work

We found that half of patients had experienced an average of 30 days of absence from work over the last three months. This result corroborates that of another study that showed a mean lost time from work in the previous 30 days equal to 12.6 days [19]. Our study also shows that, for one third of patients, days away from work are unpaid. Although indirect costs (loss of productivity) were not valued in dollars, this result suggests that the personal financial burden to working patients (24.6% of participants) is even greater than estimates provided in our results. Among non-working patients, the burden can translate into unpaid work (e.g., household work, grandchildren care, volunteering). At the time of diagnosis and treatment selection, healthcare providers should discuss with patients’ out-of-pocket costs and work absence [37] to help avoid treatment non-adherence [38] and decisional regret [13].

### 4.3. Perceived Financial Burden and Associated Factors

Rather than model costs ($), it was deemed relevant to identify factors associated with the perceived burden of out-of-pocket costs since this is more tailored to the socioeconomic profile and needs of patients. Our study suggests that prostate cancer-related out-of-pocket costs represent a significant perceived financial burden not covered by the Canadian healthcare system. These results align with those of previous work conducted in Ontario (Canada) indicating that the financial burden is problematic for 20% of cancer patients, even if these patients did not live in a remote area [19].

In our study, the higher the out-of-pocket expenses and the lower the patient income, the heavier the perceived burden (independently associated). This result is confirmed by other studies [39]. Less wealthy patients should therefore be targeted to help them plan and cope with such a burden.

Having private drug insurance was also associated with a higher perceived financial burden. This result aligns with that of Gordon et al., a study conducted in Australia where the health insurance system is universal and financed by taxes [13]. Our study data does not allow us to examine further the reasons for this result and this association should be investigated in further studies. However, one possible explanation could be that some drugs, such as sildenafil for sexual dysfunction, are generally excluded by private drug insurance. Patients were thus charged for the drug as an additional amount to the insurance premium. This additional financial charge may explain our result. Awareness of which services are covered or not by private insurance and modulation of these insurances to optimize coverage is recommendable.

Another factor associated with a heavier perceived burden was low physical health-related quality of life. This result supports that of Rogers et al. [40] who evaluated the perceived economic burden among patients with brain and neck cancer. A good functional level, a good perception of general health, little physical pain and limitation could be a possible explanation for this result.

The last factor of interest was education. In fact, independently of patients’ income, those who reached a university level were less likely to perceive the burden as significant. This result corroborates that of Azhar et al. [37] showing that higher education was a predictor of a lower perceived financial burden. This finding could be explained by the generally good level of health and financial literacy among individuals with a high level of education [41,42]. In fact, people with high levels of health literacy are less likely to perceive the economic burden as significant [43]. In addition, financial literacy helps patients better manage their financial resources and better bear their cancer economic burden [44].

### 4.4. Implications

Canadian prostate cancer patients living in the remote area of Abitibi-Témiscamingue face a financial burden despite their public medical insurance program and the government’s financial support. An increase in the allocation of resources to better reflect the true expenditures and better support patients in managing such a burden is relevant. Beyond the role of government, private insurers and community organizations could help reduce this burden by providing financial support services. Such considerations must target patients with low economic status, those who have not completed post-secondary education, and those whose physical quality of life is impaired. These measures could ultimately ensure equitable access to care.

### 4.5. Strengths and Limitations

This study has several strengths, including a high participation rate, which was comparable to other studies (66.80% vs. 72.36% [14]), and the use of a pilot-tested questionnaire that included validated scales. However, it has some limitations that should be underlined. First, an information bias is possible as costs were self-reported, and participants may have misremembered the amount of money or time spent on their prostate cancer. That said, a three-month recall period was used for the measurement of out-of-pocket costs and time loss; this should reduce the bias for these variables [45]. Second, the cross-sectional nature of our study limits the assessment of causality regarding factors associated with perceived financial burden. Third, a type 2 error is possible because our study sample size was modest (recruitment was stopped when there were no more new potential participants). Furthermore, we should mention that patient and caregiver productivity loss has not been valued in dollars because of the complexity of calculating reliable and valid estimates [30,46]. Finally, our study generalizability can be affected by the unicentric nature of the study. However, patients were recruited from a health center that serves a large population (the entire regional population of 147,508 individuals).

## 5. Conclusions

Despite the public health insurance plan and financial support to access medical services, prostate cancer was found to be a costly disease from the perspective of patients living in the remote area of Abitibi-Témiscamingue. The burden of costs perceived by patients was significant for a significant proportion of them. Certain patient characteristics had an impact on this perception. More attention should be given to alleviate the burden of prostate cancer on patients living in remote areas. Conversations between health professionals and patients about the financial consequences of prostate cancer treatment are fundamental and may help address patients’ perceived financial burden. Optimizing coverage by the public health system and private insurance companies is also essential.

## Figures and Tables

**Table 1:** Sociodemographic and clinical characteristics of the study population.

Characteristics	*n* = 171 ^a^
Age (years)—Mean ± SD	68.73 ± 7.28
Min	47
Max	87
Race/Ethnicity—*n* (%)	
White	171 (100)
Country of birth—*n* (%)	
Canada	169 (98.8)
Other	2 (1.2)
Professional status—*n* (%)	
Full-time job	28 (16.3)
Part-time job	14 (8.2)
Retired	125 (73.1)
Not working	2 (1.2)
Welfare	2 (1.2)
Relationship status—*n* (%)	
In a relationship	143 (83.6)
Single	28 (16.4)
Annual family income (CAD$)—*n* (%)	
Less than $20,000	21 (12.3)
Between $20,000 and $39,999	67 (39.2)
Between $40,000 and $59,999	39 (22.8)
Between $60,000 and $79,999	20 (11.7)
Between $80,000 and $99,999	13 (7.6)
$100,000 and over	11 (6.4)
Completed education—*n* (%)	
Elementary school	32 (18.7)
High school	66 (38.6)
Professional studies	24 (14)
College	25 (14.6)
University	24 (14.1)
Drug insurance—*n* (%)	
Régie de l’assurance maladie du Québec	123 (71.9)
Private insurance	48 (28.1)
Region of residence ^b^—*n* (%)	
Urban	98 (57.3)
Rural	73 (42.7)
PSA (ng/mL)—Mean ± SD	12.04 ± 36.54
Min	1
Max	448
Gleason score—*n* (%)	
≤6	56 (32.8)
7	90 (52.6)
>7	25 (14.6)
Tumor stage—*n* (%)	
≤T2a	130 (76.5)
T2b	5 (2.9)
T2c	35 (20.6)
Missed	1 (0.6)
Comorbidity—Mean ± SD	2.57 ± 1.50
Min	1
Max	9
Time elapsed since first treatment—*n* (%)	
0–1 year	44 (25.7)
2–3 years	53 (31.0)
4–5 years	29 (17.0)
6, 7, 8 and 9 years	27 (15.8)
10 years and over	18 (10.5)
First treatment received after diagnosis—*n* (%)	
Radical prostatectomy	44 (26.2)
Radiotherapy	96 (57.1)
Active surveillance	23 (13.7)
Hormonotherapy	4 (2.3)
Others	1 (0.7)
More than one treatment—*n* (%)	
Yes	58 (34.5)
No	110 (65.5)

^a^ Missing data across presented variables is between 0.6% and 1.8%. ^b^ Urban region (10,000 inhabitants and more); rural region (less than 10,000 inhabitants) [32].

**Table 2:** Three-month out-of-pocket costs, overall and by time since first treatment.

	Subtotal Costs for Hospital, Drugs, Vitamins, Supplements, Alternative and Complementary Therapies, etc.	Subtotal Travel Costs	Subtotal Parking Costs	Subtotal Accommodation Costs	Total Costs
**Overall**					
**3-months out-of-pocket costs for all participants ($) (*n* = 171)**					
*Mean ± SD*	136.82 ± 328.80	344.19 ± 808.53	5.90 ± 18.00	29.29 ± 185.95	516.19 ± 1029.71
*Median (range)*	15 (0–2300)	88 (0–5500)	0 (0–132)	0 (0–2200)	139 (0–6148)
**3-months out-of-pocket costs for participants with non-null costs ($) (*n* = 142)**					
*Mean ± SD*	164.76 ± 354.54	414.48 ± 871.11	7.1 ± 19.54	35.27 ± 203.66	621.61 ± 1101.09
*Median (range)*	39.5 (0–2300)	115.5 (0–5500)	1.13 (0–132)	0 (0–2200)	226.66 (5.3–6148)
**By time since first treatment**					
**3-months out-of-pocket costs for patients who are in the first year or less since first treatment ($) (*n* = 44)**					
*Mean ± SD*	182.15 ± 376.69	472.33 ± 968.68	4.75	67.95 ± 337.36	727.19 ± 1237.80
*Median (range)*	31 (0–1900)	137.5 (0–5500)	0 (0–90)	0 (0–2200)	281 (0–5790)
**3-months out-of-pocket costs for patients who are in the second year since first treatment ($) (*n* = 22)**					
*Mean ± SD*	106.40 ± 318.53	500.05 ± 946.71	11.81 ± 31.23	36.36 ± 121.67	654.64 ± 1090.12
*Median (range)*	12.5 (0–1500)	117.70 (0–4070)	1 (0–132)	0 (0–500)	221.10 (0–4110)
**3-months out-of-pocket costs for patients who are in the third year or more since first treatment ($) (*n* = 105)**					
*Mean ± SD*	124.19 ± 310.36	257.82 ± 692.83	5.13 ± 15.56	11.6 ± 74.25	398.76 ± 907.15
*Median (range)*	12 (0–2300)	33 (0–4510)	0 (0–125)	0 (0–700)	110 (0–6148)

**Table 3:** Factors associated with the perceived financial burden of prostate cancer out-of-pocket costs.

Factors	None or Slight Burden (*n* = 133)	Manageable, Difficult to Manage or Unmanageable Significant Burden (*n* = 38)	Univariate Logistic Regression *p*-Value	Crude OR (95%CI)	Adjusted OR (95%CI) ^f^
Age—Mean ± SD	69.13 ± 7.03	67.32 ± 8.02	0.18	0.97 (0.92–1.02)	
Annual family income—*n* (%) ^a^ Less than $39,999 _(reference)_ Between $40,000 and $79,999 $80,000 and over	63 (47.4) 51 (38.3) 19 (14.3)	25 (65.8) 8 (21.1) 5 (13.2)	0.04 ^e^ 0.46	0.40 (0.16–0.95) 0.66 (0.22–1.97)	0.15 (0.03–0.69) 0.09 (0.07–1.30)
Completed education—*n* (%) ^a^ Elementary and high school _(reference)_ Professional studies and college University	72 (54.1) 38 (28.6) 23 (17.3)	26 (68.4) 11 (28.9) 1 (2.6)	0.59 0.04 ^e^	0.8 (0.36–1.80) 0.12 (0.02–0.94)	0.75 (0.22–2.53) 0.03 (0.00–0.79)
Employment status—*n* (%) ^b^ Retired or not working _(reference)_ Full-time or part-time job	102 (76.7) 31 (23.3)	27 (71.1) 11 (28.9)	0.48	1.34 (0.60–3.01)	
Relationship—*n* (%) In a relationship _(reference)_ Single	112 (84.2) 21 (15.8)	31 (81.6) 7 (18.4)	0.70	1.20 (0.47–3.09)	
Region of residence—*n* (%) Urban _(reference)_ Rural	77 (57.9) 56 (42.1)	21 (55.3) 17 (44.7)	0.77	1.11 (0.54–2.30)	
Drug insurance—*n* (%) Régie de l’assurance maladie du Québec _(reference)_ Private insurance (employer- provided insurance or personally purchased insurance)	101 (75.9) 32 (24.1)	22 (57.9) 16 (42.1)	0.03 ^e^	2.30 (1.08–4.89)	5.23 (1.13–24.17)
Additional insurance—*n* (%) No _(reference)_ Yes	95 (71.4) 38 (28.6)	27 (71.1) 11 (28.9)	0.96	1.02 (0.46–2.26)	
APS at diagnosis—Mean ± SD	13.05 ± 41.28	8.51 ± 6.08	0.56	0.99 (0.96–1.02)	
Gleason score at diagnosis—Mean ± SD ^c^	6.77 ± 0.70	7.11 ± 0.76	0.01 ^e^	1.88 (1.14–3.10)	1.13 (0.53–2.40)
Clinical tumor stage at diagnosis—*n* (%) ^d^ ≤ T2a _(reference)_ T2b-T2c	100 (75.2) 33 (24.8)	30 (81.1) 7 (18.9)	0.46	0.71 (0.28–1.76)	
Time elapsed since treatment—Mean ± SD	4.44 ± 3.99	2.92 ± 2.28	0.03 ^e^	0.87 (0.77–0.99)	0.97 (0.82–1.15)
Number of treatments received—Mean ± SD	1.35 ± 0.58	1.66 ± 0.75	0.01 ^e^	2.01 (1.18–3.43)	2.02 (0.80–5.11)
Out-of-pocket costs—Mean ± SD	249.20 ± 410	1450.65 ± 1765.81	0.00 ^e^	1 (1–1)	1.001 (1.001–1.002)
Physical health-related quality of life—Mean ± SD	48.42 ± 9.31	42.14 ± 11.55	0.00 ^e^	0.95 (0.91–0.98)	0.95 (0.913–0.997)
Mental health-related quality of life—Mean ± SD	47.91 ± 8.51	41.90 ± 10.30	0.00 ^e^	0.93 (0.90–0.97)	0.947 (0.895– 1.002)
Treatment in the region vs. outside the region—*n* (%) In the region Outside the region _(reference)_	47 (36.2) 83 (63.8)	6 (16.2) 31 (83.8)	0.03 ^e^	2.93 (1.14–7.52)	0.87 (0.22–3.41)

^a^ Variable originally measured using five answer categories but was regrouped into three in logistics analyses. The recategorization was distribution-based. ^b^ Variable originally measured using five answer categories but was regrouped into two in logistics analyses. The recategorization was distribution-based. ^c^ Categorical variable originally, converted into a continuous variable in logistics analyses given the modest sample size. ^d^ Variable originally measured using three answer categories but was regrouped into two in logistics analyses. The recategorization was distribution-based. ^e^ *p*-value ≤ 0.05. ^f^ Estimate of adjusted OR for all factors with a *p*-value ≤ 0.15 in the univariate logistic regression models and time elapsed since treatment.
